# SNP-SNP interactions in breast cancer susceptibility

**DOI:** 10.1186/1471-2407-6-114

**Published:** 2006-05-03

**Authors:** Venüs Ümmiye Onay, Laurent Briollais, Julia A Knight, Ellen Shi, Yuanyuan Wang, Sean Wells, Hong Li, Isaac Rajendram, Irene L Andrulis, Hilmi Ozcelik

**Affiliations:** 1 Fred A. Litwin Centre for Cancer Genetics, Samuel Lunenfeld Research Institute, Mount Sinai Hospital, Toronto, Ontario, Canada; 2 Prosserman Centre for Health Research, Samuel Lunenfeld Research Institute, Mount Sinai Hospital, Toronto, Ontario, Canada; 3 Department of Pathology and Laboratory Medicine, Mount Sinai Hospital, Toronto, Ontario, Canada; 4 Ontario Cancer Genetics Network, Cancer Care Ontario, Toronto, Ontario, Canada; 5 Department of Public Health Sciences, University of Toronto, Toronto, Ontario, Canada; 6 Department of Molecular and Medical Genetics, University of Toronto, Toronto, Ontario, Canada; 7 Department of Laboratory Medicine and Pathobiology, University of Toronto, Toronto, Ontario, Canada

## Abstract

**Background:**

Breast cancer predisposition genes identified to date (e.g., BRCA1 and BRCA2) are responsible for less than 5% of all breast cancer cases. Many studies have shown that the cancer risks associated with individual commonly occurring single nucleotide polymorphisms (SNPs) are incremental. However, polygenic models suggest that multiple commonly occurring low to modestly penetrant SNPs of cancer related genes might have a greater effect on a disease when considered in combination.

**Methods:**

In an attempt to identify the breast cancer risk conferred by SNP interactions, we have studied 19 SNPs from genes involved in major cancer related pathways. All SNPs were genotyped by TaqMan 5'nuclease assay. The association between the case-control status and each individual SNP, measured by the odds ratio and its corresponding 95% confidence interval, was estimated using unconditional logistic regression models. At the second stage, two-way interactions were investigated using multivariate logistic models. The robustness of the interactions, which were observed among SNPs with stronger functional evidence, was assessed using a bootstrap approach, and correction for multiple testing based on the false discovery rate (FDR) principle.

**Results:**

None of these SNPs contributed to breast cancer risk individually. However, we have demonstrated evidence for gene-gene (SNP-SNP) interaction among these SNPs, which were associated with increased breast cancer risk. Our study suggests cross talk between the SNPs of the DNA repair and immune system (XPD-[Lys751Gln] and IL10-[G(-1082)A]), cell cycle and estrogen metabolism (CCND1-[Pro241Pro] and COMT-[Met108/158Val]), cell cycle and DNA repair (BARD1-[Pro24Ser] and XPD-[Lys751Gln]), and within carcinogen metabolism (GSTP1-[Ile105Val] and COMT-[Met108/158Val]) pathways.

**Conclusion:**

The importance of these pathways and their communication in breast cancer predisposition has been emphasized previously, but their biological interactions through SNPs have not been described. The strategy used here has the potential to identify complex biological links among breast cancer genes and processes. This will provide novel biological information, which will ultimately improve breast cancer risk management.

## Background

The existence of dominant predisposition alleles/mutations, conferring a high breast cancer risk, has been confirmed with the discovery of BRCA1 and BRCA2 [1,2]. The functionally defective mutations of BRCA1 and BRCA2 are strongly associated with dramatically increased breast cancer risk, however, such mutations are found to be rare (<5%) in unselected breast cancer cases [1-3]. Besides these rare mutations, commonly occurring single nucleotide polymorphisms (SNPs) have also been shown to incrementally contribute to breast cancer risk, however, their individual contributions are relatively small [4-6].

SNPs have been historically classified as commonly occurring (>1%) genetic variation in the general population, whereas the rare variants with obvious functional consequences on the protein have been classified as mutations. Compared to mutations, SNPs have been perceived as functionally insignificant, however, current evidence emphasizes that a considerable fraction affects the intrinsic properties and the function of the proteins to a variable degree [7-9]. Although the effect of an individual SNP is generally small, the genetic effect of combinations of functionally relevant SNPs may additively or synergistically contribute to increased breast cancer risk. Epistasis or gene-gene interaction is likely to be a ubiquitous component of the genetic architecture of common diseases, such as breast cancer. The effects of epistasis could dictate functional outcomes over the independent effects of any one susceptibility gene [10]. Polygenic models have also been proposed to explain the joint effect of many susceptibility alleles on breast cancer, but without considering specifically their possible interactions [11-14].

To estimate breast cancer risk conferred by individual SNPs, as well SNP-SNP interactions, we have studied 19 SNPs from 18 key cancer genes, involved in DNA repair (XPD, PTEN, GADD45), cell cycle (CCND1, p27, BARD1), carcinogen/estrogen metabolism (ESR1, CYP17, COMT, GSTP1, GSTM3, MTHFR), immune system (IL1a, IL10, IL13, TNFa, G-CSF) and others (MMP1) (Table 1). SNPs were initially selected from the best evidence from published studies in the beginning of the project, in year 2000, and subsequently classified under three categories (high-, medium- and low- rank), representing SNPs with a wide range of functional evidence. High-rank SNPs were supported by studies, which demonstrated the effect of the SNP on the regulation of expression or protein function. The medium-rank category is more likely to include functionally relevant SNPs, as the substitutions are predicted to significantly affect function, although this was not confirmed experimentally. This category also includes SNPs, which were associated with breast cancer risk factors. The low ranking category, on the other hand, contained SNPs with no functional information. Among the SNPs studied, XPD-[Lys751Gln], MTHFR-[Ala222Val], COMT-[Met108/158Val], GSTP1-[Ile105Val] and CCND1-[Pro241Pro], have been shown to alter the function or post-translational modification of their encoded protein [15-27]. MMP1-[1G(-1607)2G] and IL10-[G(-1082)A] have been shown to alter the transcription and expression of these genes [28-32]. IL13-[Arg130Gln] has been suggested to have functional consequences, while GSTM3-[4595 (3bp ins/del)] was predicted to create a YY1 transcription factor binding site [33,34]. The TNFA-[G(-308)A] forms a haplotype with some nearby SNPs and some studies observed increased haplotype dependent transcriptional activity change while some others do not [35-38]. CYP17-[C518T], and IL13-[Arg130Gln] were found to be associated with other cancer related variables, such as serum estrogen and IgE levels, respectively [39-41]. BARD1-[Pro24Ser] changes a structurally important non-polar proline residue to a positively charged serine. There were no functional speculations for ESR1-[Ser10Ser], ESR1-[Pro325Pro], PTEN-[(IVS4+109)ins/delACTAA], IL1A-[Ala114Ser], G-CSF-[Leu185Leu]and GADD45-[C(IVS3+168)T. Thus, the 19 SNPs studied represent SNPs with a wide range of functional knowledge and evidence. The range of the minor allele frequencies of 19 SNPs studied varied between 15-48% in the general population. The SNPs studied were selected to represent more commonly occurring variants, in order to gain statistical power to detect SNP-SNP interactions.

## Methods

### Subject population

A case control study was conducted using biospecimens and data from the Ontario Familial Breast Cancer Registry (OFBCR) a participating site in the NIH-funded Breast Cancer Family Registry [42]. Written informed consent was obtained from all subjects, and the study protocol was approved by Mount Sinai Hospital Research Ethics Board.

Cases of invasive breast cancer, pathologically confirmed and diagnosed between 1996 and 1998 in the province of Ontario were identified from the population-based Ontario Cancer Registry. All female cases under 55, a random sample (35%) of female cases aged 55 to 69, and all male cases under age 80 were identified. Physician permission to contact patients was granted for 91% of cases (7668 of 8453). Patients were then mailed a cancer family history questionnaire and 65% (4957) completed it. All respondents who met a defined set of genetic risk criteria (i.e., Ashkenazi Jewish; diagnosed before age 36 years; previous ovarian or breast diagnosis; one or more first- or two or more second-degree relatives with breast or ovarian cancer; one or more second- or third-degree relatives with either breast cancer diagnosed before age 36 years, ovarian cancer diagnosed before age 61 years, multiple breast or breast and ovarian primaries, or male breast cancer; three or more first-degree relatives with any combination of breast, ovarian, colon, prostate, or pancreatic cancer or sarcoma, with at least one diagnosis before age 51 years) were included in the study [43] and a random sample of 25% of those not meeting criteria were selected to continue to participate in the OFBCR (n= 2580). This participation included providing a blood sample (provided by 62% of all eligible, n = 1601). For the current study, we restricted the sample to women who identified themselves as Caucasian and were less than 55 years old. As we had randomly sampled 25% of those who did not meet genetic risk criteria, we also randomly sampled 25% of those who did meet genetic risk criteria in order to create a more representative sample of cases. Therefore, the cases should better represent all cases without enrichment for genetic risk criteria such as family history. In Table 2, 21.6% of cases in the present study had a first-degree family history of breast cancer, which is consistent with the 17 to 22% frequency reported in cases in a number of large case-control studies [44-46]. Of 459 Caucasian breast cancer cases with blood available, 398 were successfully genotyped and included in the study.

Controls were identified by calling randomly selected residential telephone numbers from across the province of Ontario and were frequency-matched to all female OFBCR cases by 5-year age group. The number of telephone numbers was 14,653, but 1101 (8%) were invalid and no contact could be made for 841 (6%). Of the 12,711 households contacted, 7829 (62%) did not have an eligible individual. No information on eligibility was provided for 2194 (17%) households. Of the 2688 eligible individuals identified on the telephone, 1726 (64%) completed the mailed risk factor questionnaire and 75% of these agreed to be contacted about providing a blood sample. The 676 women under age 55 who had agreed to be approached about blood sampling were asked to provide a blood sample and 419 (62%) did so. Individuals who were not Caucasian were excluded from the analysis, as were those with insufficient DNA or those subsequently found to be ineligible because of age. The remaining 372 population controls were successfully genotyped in this study.

### Molecular genotyping

All SNPs were analyzed by TaqMan 5'nuclease assay [47] using the ABI PRISM 7900 HT Sequence Detection System (version 2.0). Oligonucleotide primers and the dual labeled allele specific probes were designed using PrimerExpress version 2.0 (PE Biosystems). Positions of primers for and probes in their appropriate accession numbers are given in Additional file 1.

A panel of DNA samples were sequenced for each SNP region initially, in order to identify control genotypes to be used in each experiment. PCRs were performed in 96 well plates (AXYGEN) with each plate containing four control samples for each possible genotype. Genomic DNA (10 ng) was amplified in a total volume of 10 ul in the presence of 100 uM of each of the dNTPs, 3 pmoles of each of the appropriate primers, 2 pmoles of each of the corresponding dual labelled probes, and 0.025 units of Platinum Taq DNA Polymerase (InVitrogen). PCR cycling conditions consisted of 40 cycles of 94°C for 15 sec, 55–60°C for 15 sec and 72°C for 15 sec. The optimal MgCl_2 _concentrations and annealing temperatures for each SNP are given in Additional file 2. The reliability of the results was determined by re-genotyping a randomly selected 10% portion of the total study population.

### Statistical analyses

We sought evidence of association between each of the 19 SNPs and breast cancer risk in a multi-step process. At the first stage, we calculated crude allele and genotype frequencies for each individual polymorphism and evaluated Hardy-Weinberg equilibrium using a one-degree of freedom goodness-of-fit test among controls [48]. The association between the case-control status and each individual SNP, measured by the odds ratio (OR) and its corresponding 95% confidence interval, was estimated using unconditional logistic regression after adjustment for age. Several epidemiological risk factors were also assessed for association with breast cancer including age, BMI, education status, smoking status, family history, menopausal status, age at menarche, age at menopause, parity and age at first live birth (Table 2). Some of our analyses were also carried out adjusting the SNP main effect for the statistically significant epidemiological risk factors.

All analyses were performed assuming a dominant, recessive and co-dominant effect for each polymorphism. In the dominant model, both the heterozygous variant and the rare homozygous variant were combined. In the recessive model, the variant was defined as only the rare homozygous genotype and in the co-dominant model both rare homozygous and heterozygous variant effects were estimated using two dummy variables. In all analyses, the common homozygote genotype in the control population was defined as the reference category. Age was considered as a continuous variable. The likelihood ratio test was used to test the effect of each SNP at the nominal 5% significance level. Akaïke's information criterion [49] was also used to select the best genetic effect for each SNP.

At the second stage, two-way interactions were investigated using multivariate logistic models. More specifically, we tested all SNP-SNP interactions. We assumed a multiplicative interaction effect on the logit scale. Statistically significant interactions were selected using a forward stepwise selection procedure to evaluate evidence that specific interactions were independently associated with breast cancer. The initial model included all SNPs and age as main effects, and then searched for the most significant candidate interactions to enter into the model based on the score statistics at the 5% level. Backward elimination of variables was then performed using the likelihood ratio test (LRT) also at the level of 5%. Forward stepwise selection procedure has proven to be efficient in assessing interaction effects as compared to backward elimination when testing multiple interactions. First, it is more time efficient and second, when using backward elimination, a relatively large number of predictor variables may increase the risk of complete separation of the two outcome groups, which would result in numerical problems in estimating the model parameters [50]. Since the genetic risk model is uncertain for most of the SNPs considered, we performed these tests on the co-dominant models only. Therefore, tests for SNP-SNP interactions have four degrees of freedom. All these analyses were also performed adjusting the interaction effects for the risk factors found to be associated with breast cancer risk at a significance level of 5% (BMI and family history).

We have also estimated the amount of linkage disequilibrium (LD) between the two ESR1 SNPs separated by about 140 kb on chromosome 6 and investigated their haplotype effect on breast cancer using the software "Unphased" from Dudbridge [51].

The large number of interactions (n = 171) analyzed could lead to false positive results, therefore, we adopted two different strategies to avoid this problem: The first approach included the assessment of the selection procedure using bootstrapping and the second one included an adjustment for multiple testing using detection rate (FDR). The bootstrap approach selects random samples of size *n *(*n*_1 _cases + *n*_2 _controls) with replacement from the original data [52]. Repeating the sampling procedure a large number of times provides information on the variability and validity of the parameter estimate and model selection. We repeated the selection procedure on 1,000 random samples (each random sample comprising 398 cases and 372 controls), generated from the original sample and the number of times a particular interaction was selected was reported. The achieved significance level (ASL) from the bootstrap test of hypotheses was also computed. Following Efron and Tibshirani [52], the ASL was obtained by comparing the observed LRT statistic for a specific interaction to its null distribution, evaluated by randomly assigning the case-control status in 1,000 bootstrap samples. The second approach tries to correct formally for the multiple testing problem using the FDR principle [53]. This procedure does not control the experiment-wise error rate like the Bonferroni-type correction (which is known to be conservative) but estimates the proportion of errors among the rejected null hypotheses. FDR was applied to both main effect models and interaction models using bootstrap *P*-values. For these latter models, bootstrap *P*-values and FDR-adjusted *P*-values correspond to interaction effects in multivariate logistic models that include all main effects and only the interaction of interest. This is equivalent to the test performed at the first step of the forward stepwise regression. We also computed the probability of no true association between an interaction and the disease status given a statistically significant result (i.e. the false positive report probability, FPRP) proposed by Wacholder et al. [54]. This statistic depends on the observed *P*-value but also on both the prior probability that the association between the SNP-SNP interaction and the disease is real and the statistical power of the test. The power of the test was determined by computing the expected value of the likelihood ratio test statistic, assuming our data were analyzed by the unconditional age-adjusted logistic model and with the specific coding of the SNP-SNP interactions using four dummy variables (see above). This computation was implemented into an R program, following the method described by Gauderman [55,56]. We used informative prior probabilities using the functional studies presented in Table 1 to classify the importance of each SNP. Following Wacholder's recommendations [54], the probability assigned to each SNP was 0.10, 0.01 and 0.001 for the high-, medium-, and low-ranked SNPs respectively. The prior joint probability for each pair of SNPs was just the product of the individual SNP probability. Finally, we used the bootstrap *P*-values in the FPRP computation.

## Results

Table 1 gives the minor allele frequencies of 19 SNPs estimated in our control population. None of the SNP distributions showed deviation from Hardy-Weinberg equilibrium in this sample. The distribution of selected epidemiologic risk factors in cases and controls is shown in Table 2. Cases and controls were similar with respect to the distribution of smoking status, menopausal status, age at menarche, age at menopause, parity, and age at first birth. Controls tended to have a higher BMI (*p *= 0.05) and level of education (*p *= 0.06) than cases. Cases were also more likely to have a positive family history of breast cancer than controls, and this difference was highly significant (*p *= < 10^-5^). Logistic regression analysis was performed for all SNPs in the context of recessive, dominant and co-dominant models after adjustment for age. The estimated ORs and 95% CIs for all SNPs under co-dominant models are shown in Table 3.

Among the 19 SNPs studied, XPD-[Lys751Gln] was the only one showing a significant main effect in our sample based on the crude *P*-value. However, after correction for multiple testing using FDR, the effect was not significant. Our results remained unchanged when the models were also adjusted for BMI and family history. Results of two-way interaction analyses are shown in Table 4. Since the genetic risk models are uncertain for most of the SNPs considered, we performed these tests on the co-dominant models only. A total of nine SNP-SNP interactions were consistently selected in at least 30% of the random samples by the stepwise procedure. Interactions were observed more frequently for XPD-[Lys751Gln] and IL10-[G(-1082) (68%), and COMT-[Met108/158Val] and CCND1-[Pro241Pro] (61%). Interactions between GSTP1-[Ile105Val] and COMT-[Met108/158Val], CYP17-[C(518)T] and GADD45-[C(IVS3+168)T], and BARD1-[Pro24Ser] and ESR1-[Pro325Pro] selected in 54%, 53% and 51% of the random samples, respectively. All interactions, except BARD1-[Pro24Ser] and ESR1-[Pro325Pro], and, BARD1-[Pro24Ser] and p27-[Val109Gly], were statistically significant (P < 0.05) based on the bootstrap *P*-values.

After correction for multiple testing using FDR principle, four interactions remained significant at the 5% level; XPD-[Lys751Gln] and IL10-[G(-1082)A] (p = 0.007), GSTP1-[Ile105Val] and COMT-[Met108/158Val] (p = 0.007), COMT-[Met108/158Val] and CCND1-[Pro241Pro] (p = 0.014), and BARD1-[Pro24Ser] and XPD-[Lys751Gln] (p = 0.014). Based on the False Positive Report Probability (FPRP) approach, computed using the functional importance of each SNP, we found that three interactions were noteworthy at the 0.2 FPRP level (Table 4); XPD-[Lys751Gln] and IL10-[G(-1082)A] (FPRP = 0.092), GSTP1-[Ile105Val] and COMT-[Met108/158Val] (FPRP = 0.169), and COMT-[Met108/158Val] and CCND1-[Pro241Pro] (FPRP = 0.093). The effect size of each SNP-SNP genotype combination for the four significant interactions is given in Table 5.

None of these interactions were significant after the more conservative Bonferroni adjustment. The Bonferroni adjusted *P*-values were 0.19 for both XPD-[Lys751Gln] and IL10-[G(-1082)A], and GSTP1-[Ile105Val] and COMT-[Met108/158Val] interactions. The *P*-values for the COMT-[Met108/158Val] and CCND1-[Pro241Pro], and BARD1-[Pro24Ser] and XPD-[Lys751Gln] interactions were 0.38.

The results of our multivariate analyses adjusted for age, BMI and family history confirmed the role of the most important interactions. The bootstrap *P*-values associated with XPD-[Lys751Gln] and IL10-[G(-1082)A], COMT-[Met108/158Val] and CCND1-[Pro241Pro], GSTP1-[Ile105Val] and COMT-[Met108/158Val], and BARD1-[Pro24Ser] and XPD-[Lys751Gln] were all significant (respectively, *P *= 0.014, *P *= 0.020, *P *= 0.022 and *P *= 0.020), however the significance of the tests decreased due to the high proportion of individuals missing BMI information. Therefore, only the analyses adjusted for age are presented in Table 4.

The amount of LD between the two ESR1 SNPs was relatively small, with a D' [57] of 0.07 in cases and 0.15 in controls, and none of the four haplotypes was significantly associated with breast cancer. Therefore, only the interaction effect between the two SNPs is presented.

## Discussion

In this study, we have analysed the contribution of 19 SNPs from 18 cancer-related genes, to breast cancer risk in a case-control study of 398 breast cancer cases and 372 population controls, sampled from the population-based OFBCR. All cases and controls were Caucasian women under age 55. We found that among 19 SNPs, XPD-[Lys751Gln] substitution was the only one showing a significant association with breast cancer risk. However, after correction for multiple testing, the effect became insignificant, suggesting that this finding might be due to chance. Overall we found little evidence of breast cancer risk conferred by individual commonly occurring SNPs in this dataset.

Our main focus was to understand the contribution to breast cancer risk of functionally relevant SNP-SNP interactions within and between different cancer pathways. Recently, there has been increasing evidence regarding the joint effect of commonly occurring SNPs on cancer risk, supported by polygenic breast cancer models [11-14]. Although this model was originally based on the additive effects of multiple risk alleles [14,58] (each with a low to moderate risk) it can be generalized by considering interaction effects between the susceptibility alleles.

In this study, we have shown several statistically significant interactions between SNP pairs. Despite the low false discovery rates observed for certain interactions and small-unadjusted *P*-values, caution should be used when interpreting these results. First, the statistical modeling of interactions through a product term might not correspond to or reflect any biological interaction such as synergy or antagonism [59,60]. Second, as in any association study, epidemiologic limitations such as selection bias or confounding have the potential to lead to false-positive results. Cases were selected from a population-based cancer registry and although selection may have occurred, there was no evidence of selection related to family history of breast cancer [43,61]. This analysis was also restricted to Caucasians who had the highest response rates and also minimizes the potential for population stratification.

To assess the robustness of interaction models, we used two different strategies: an internal validation procedure based on bootstrap re-sampling methods [52] and a correction for multiple testing using the FDR principle [53]. The first approach allowed us to prioritize nine "candidate" SNP-SNP interactions that were consistently selected by the stepwise procedure across the bootstrap samples (i.e. in more than 30% of the 1,000 random samples) and that were significant based on the age adjusted bootstrap *P*-values. This method can be used to assess the variability of our model selection but does not control formally for the multiple testing problem. Although correction for multiple testing can be performed under the bootstrap framework [62] or using the familiar Bonferroni correction, these procedures can be very conservative. We therefore used the FDR principle that does not control the experiment-wise error rate but estimates the proportion of errors among the rejected null hypotheses. Using this correction, none of the SNP main effects were significant, but four 2-way interactions had adjusted *P-v*alues lower than 5%. The stepwise selection procedure applied to our original data set without validation or correction would have detected 14 significant SNP-SNP interactions at the 5% level. Based on our validation procedures, ten of these are likely to be false positive results. This shows the importance of model validation in studies of gene interactions [63]. Although this validation was internal (i.e. using the same data set), we intend to replicate our results using an external data set.

To interpret our positive results, we should also remember that our SNPs were selected from genes involved in cancer, and enriched by SNPs that are likely to affect the function of the encoded protein. Using a Bayesian approach, Wacholder [54] recently showed how the probability of no true association between a genetic variant and disease given a statistically significant result (i.e. the false positive report probability (FPRP) depends on the prior probability that the association is real and also on the statistical power of the test. Although the determination of a prior probability is quite challenging [64], selecting SNPs based on their functions clearly reduce the FPRP. Based on this approach, we found that three out of the four significant interactions were noteworthy at the 0.2 FPRP level: XPD-[Lys751Gln] and IL10-[G(-1082)A], GSTP1-[Ile105Val] and COMT-[Met108/158Val], and COMT-[Met108/158Val] and CCND1-[Pro241Pro]. More complex approaches to account for the prior knowledge of the functional importance of each SNP have also been proposed [65-68]. We intend to use some of these methods to confirm our results in future analyses. Another problem to consider is the chance for false negative results. SNPs that were considered as negative after our conservative multiple comparison adjustment might still be worthy of investigation in other data sets. Therefore, some of our results must be viewed as hypothesis generating. Validation of our results (positive and negative) in an independent data set will provide further insight into the role of these SNPs in breast cancer etiology.

The novelty of our study is the demonstration of statistically significant interactions between SNPs that did not have an effect on breast cancer risk individually. Most studies have investigated the main effects of commonly occurring SNPs and categorized them as "not associated", and thus not important in breast cancer risk. However, our study suggests that SNPs without main effects or with main effects, too small to detect, may interact with others and confer an increased risk for breast cancer. Larger studies will allow a better application of our model, in which more complex interactions could be investigated. SNP-SNP interactions in breast cancer development have been also reported in other studies, which targeted the SNPs of the carcinogen metabolism genes, including GSTM1, GSTT1, GSTP1, GSTM3 and CYPs [69-73]. These reports support our findings regarding SNP-SNP interactions on breast cancer risk, although they were only limited to the interactions of SNPs within a single cancer pathway.

In the context of breast cancer predisposition, our study suggests there is cross talk between the alleles of proteins of different cancer pathways including DNA repair and the immune system (XPD-[Lys751Gln] and IL10-[G(-1082)A]), cell cycle and estrogen metabolism (COMT-[Met108/158Val] and CCND1-[Pro241Pro]), cell cycle and DNA repair (BARD1-[Pro24Ser] and XPD-[Lys751Gln]) as well as within a single pathway such as estrogen metabolism (GSTP1-[Ile105Val] and COMT-[Met108/158Val]). Among the four interactions identified, COMT and XPD seem to play a central role since they interacted with different proteins in our set. XPD interaction was identified with both IL-10 and BARD1; whereas COMT interaction was identified with CCND1 and GSTP1. Estimation of odds ratios for particular genotype combinations can show considerable increase in the risk associated with breast cancer. For example the combination of rare genotypes of BARD1-[Ser24] (TT genotype) and XPD-[Gln751] (CC genotype) resulted in a relative risk of 7.4 (95% CI: 1.3-12.4). This is an excellent example where rare genotype combinations are associated with increased cancer risk. Such findings would have been missed in the absence of interaction analysis. These results emphasize the need for larger studies where the risk associated with such rare genotype combinations can be validated.

**Figure 1:**
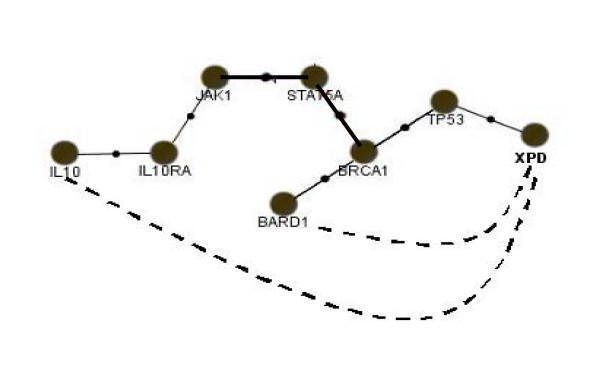
**Evaluation of statistical interactions of XPD with IL-10 and BARD1 on the bases of protein-protein interactions of their intermediate proteins**. Solid lines and dashed lines are used wherever there is a protein-protein or a statistical interaction, respectively. The protein-protein interaction map has been obtained using a protein-protein interaction database [82].

**Figure 2:**
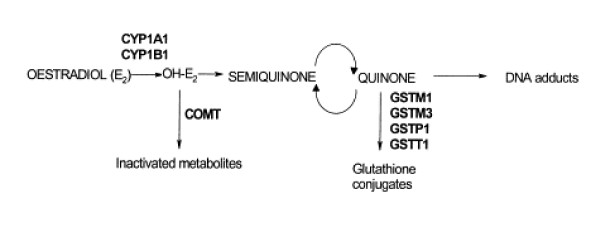
**A simplified drawing showing the roles of COMT and GSTP1 enzymes in estrogen metabolism**. The figure is modified from [4] with permission.

We have further investigated the biological relationships between XPD and COMT interactions with other SNPs using protein-protein interaction databases and a literature search. As seen in Figure 1, statistical interaction between XPD-[Lys751Gln] and BARD1-[Pro24Ser] can be accounted for by protein-protein interactions among ERCC2 (XPD), TP53, BRCA1 and BARD1 [74,75]. Similarly the statistical interaction between XPD-[Lys751Gln] and IL10-[G(-1082)A] can be accounted for by protein-protein interactions among XPD(ERCC2), BRCA1, TP53, STAT5A, JAK1, IL-10RA and IL-10 [74-77]. It is important to point out that interaction of XPD (ERCC2) with IL-10 and BARD1 revolves around the protein-protein interaction of BRCA1 and TP53. As shown previously, mutations in these two proteins lead to predisposition to hereditary breast cancer [1,78]. This supports the observation that SNPs of XPD (ERCC2), BARD1 and IL-10 may be good candidates for breast cancer predisposition, which may also modify the effect of BRCA1 in carriers. On the other hand, statistical interaction of COMT-[Met108/158Val] with GSTP1-[Ile105Val] and CCND1-[Pro241Pro] revolves around estrogen metabolism and cell proliferation (Figure 2). Estrogen is an important risk factor for breast cancer. Estrogen is broken down into reactive species by phase I enzymes, which are then inactivated by phase II enzymes such as the methylation of catechol estrogens by COMT or the conjugation of oxidized estrogen-quinones by GSTP1. The relation between COMT and GSTP presumably depends on reduced inactivation of the aforementioned reactive estrogen intermediates, because of decreased activities of both enzymes [4,22,23]. The regulated level of estrogen might in turn influence the cell proliferation through CCND1 transcription [4,24-27,79].

The biological pathways investigated in this study have been previously implicated in breast cancer development; however, their genetic interactions, detected through variant alleles (SNPs), have not been previously described. These data and the statistical approaches applied to them have the potential to assist in the identification of complex biological relationships among cancer processes during the development of breast cancer. When moving into the era of "genetic dissection of complex traits [80]", we will need to abandon the concept of single genetic determinants to favor the idea of a "web of causation [81]" involving multiple and complex pathways, which in turn could implicate many genes and environmental factors. This study provides a possible framework for a functional SNP-SNP interaction-based model for breast cancer risk.

## Conclusion

Our focus in this study has been to uncover SNP-SNP interactions, which additively or synergistically contribute to breast cancer risk. From our small pool of SNPs, we have shown significant statistical interactions suggesting biological cross talk among genes/SNPs from DNA repair, cell cycle, immune system and carcinogen metabolism pathways. Our immediate task is to apply this strategy to a larger sample, with the aim of replicating our findings and investigating more complex interactions (involving three or more SNPs). This line of research has the potential to identify important cross talk between members of the cancer pathways in the disease state. This study not only provides insight into the analysis of the multi-genic nature of breast cancer, but also provides important information regarding how cell function relates to breast cancer development. We believe that these and other interactions in breast cancer will one day be identified and used in clinics to identify individuals at increased risk of breast cancer and develop preventive strategies.

## Abbreviations

**SNP **– single nucleotide polymorphisms

**FDR **– false discovery rate

**FPRP **– false positive report probability

**OFBCR **– Ontario familial breast cancer registry

**OR **– odds ratio

**LRT **– likelihood ratio test

**ASL **– achieved significance level

**LD **– linkage disequilibrium

## Competing interests

The author(s) declare that they have no competing interests.

## Authors' contributions

VUO participated in design of the study, in data production and management, and manuscript preparation.

LB carried out the statistical analyses and wrote of the statistical sections of the manuscript.

JAK participated in formation of epidemiological study design and manuscript preparation.

ES participated in statistical analysis.

YW participated in statistical analysis.

SW participated in production of genotype data.

HL participated in production of genotype data.

IR participated in statistical analysis.

ILA provided information about study samples and OFBCR, and participated in manuscript preparation.

HO participated in the design and coordination of the study, and manuscript preparation.

All authors read and approved the final manuscript.

## Pre-publication history

The pre-publication history for this paper can be accessed here:



## Supplementary Material

Additional file 1 Primer_and_probe_information_for_19_SNPs. Positions of primers and probes used in the study, in their appropriate accession numbers, are given in this table. Click here for file

Additional file 2 PCR_Conditions_for_19_SNPs. The optimal MgCl_2 _concentrations and annealing temperatures for each SNP PCR are given in this table. Click here for file

## Figures and Tables

**Table 1:** SNPs selected for the study and their functional consequences.

**SNPs**	**Minor Allele (%)**	Interacting SNPs*	**Alteration**	**Function**	**Breast Cancer Association§**	**Selected Biological Processes¶**
***Higher Rank (Direct Functional Evidence)***						

XPD-[Lys751Gln]	C: 32.3	Yes	Missense	Gln (C) allele has decreased DNA repair capacity [15-18]	Yes [83]	transcription-coupled nucleotide-excision repair, ATP-dependent DNA helicase activity
COMT-[Met108/158Val]	G: 47.3	Yes	Missense	Met (A) allele has lower enzymatic activity [20,21]	Yes [84]	neurotransmitter catabolism, catecholamine metabolism
GSTP1-[Ile105Val]	G: 31.2	Yes	Missense	Val (G) allele is associated with reduced enzymatic activity [22,23]	Yes [85]	Metabolism, glutathione transferase activity
MTHFR-[Ala222Val]	T: 35.6	No	Missense	Val (T) allele is associated with reduced enzyme activity [19]	Yes† [86]	Folate metabolism, methylene-tetrahydrofolate reductase (NADPH) activity
CCND1-[Pro241Pro]	A: 46.1	Yes	Splice Variant	(A) allele affects protein stability [24-27]	Yes [87]	G1/S transition of mitotic cell cycle
MMP1-[1G(-1607)2G]	Ins: 49.7	No	Regulatory	(Ins) allele shows increased transcription [28,29]	No	collagen catabolism, interstitial collagenase activity
IL10-[G(-1082)A]	G: 47.6	Yes	Regulatory	G allele is associated with increased expression [30-32]	No	cell-cell signaling, B-cell differentiation and proliferation, anti-apoptosis.

***Medium Rank (Indirect Functional Evidence)***						

BARD1-[Pro24Ser]	T: 37.5	Yes	Missense	Proline to serine change is a significant alteration	No	response to DNA damage, protein ubiquitination, regulation of apoptosis
IL13-[Arg130Gln]	A : 18.3	No	Missense	Gln allele associated with increased IgE levels [41]	No	immune response, inflammatory response, signal transduction,
p27-[Val109Gly]	G: 19.4	No	Missense	Possible function [33]	No	regulation of cyclin dependent protein kinase activity
GSTM3-[4595 (3bp ins/del)]	Del: 16.8	No	UTR	creates recognition site for transcription factor YY1 [34]	No	Metabolism, glutathione transferase activity
TNFA-[G(-308)A]	A: 17.6	No	UTR	increased transcriptional activity; also no functional change [35-38]	No	inflammatory response, signal transduction, regulation of transcription, apoptosis
CYP17-[C(518)T]	C: 34.3	No	Regulatory	Associated with increased serum estradiol [39,40]	Yes [4]	C21-Steroid hormone metabolism,

***Lower Rank (No Functional Evidence)***						

IL1A-[Ala114Ser]	T : 27.4	No	Missense	no published functional evidence	No	MAPK signaling pathway, regulation of progression through cell cycle
GADD45-[C(IVS3+168)T]	C: 31.2	No	Intronic	no published functional evidence	No	regulation of cyclin dependent protein kinase activity, DNA repair, apoptosis
PTEN-[(IVS4+109)ins/del5	Ins: 30.1	No	Intronic	no published functional evidence	No	negative regulation of cell cycle, protein tyrosine/serine/threonine phosphatase activity
ESR1-[Ser10Ser]	C: 48.5	No	Silent	no published functional evidence	No	steroid hormone receptor activity, signal transduction, regulation of transcription
G-CSF-[Leu185Leu]	G: 38	No	Silent	no published functional evidence	No	immune response, cell-cell signaling, positive regulation of cell proliferation
ESR1-[Pro325Pro]	G: 24.1	No	Silent	no published functional evidence	No	steroid hormone receptor activity, signal transduction, regulation of transcription

*No and Yes indicates whether the SNP has been shown to be "interacting" or "not interacting" with other SNPs in this study; **§ **The studies that showed statistically significant overall SNP-disease risk associations were considered (N_cases _> 250 and N_controls _> 250).

†Cases are women diagnosed with breast cancer before age 40; ¶ GeneCards [88]

**Table 2:** Baseline characteristics of breast cancer cases and controls.

	**Cases**		**Controls**		***P *value**^#^
	
	***n***	**%**	***n***	**%**	
**Overall**	398		372		

Age*					
≤ 40	79	19.8	69	18.6	0.08
41–45	106	26.6	98	26.4	
46–50	144	36.2	113	30.4	
51–55	69	17.3	91	24.5	
Missing	0	-	1	-	
Mean (SD)	44.8	(6.2)	45.2	(6.6)	0.37

***BMI***					
<18.5	7	2.2	4	1.1	0.05
18.5 – 25	178	55.1	166	46.0	
25 – 30	81	25.1	116	32.1	
> 30	57	17.6	75	20.8	
Missing	75	-	11	-	
Mean (SD)	25.7	(5.4)	26.3	(5.5)	0.14

***Education***					
Up to high school	122	35.4	101	27.2	0.06
Technical school	27	7.8	34	9.2	
Some college/University	196	56.8	236	63.4	
Missing	53	-	1	-	

***Smoking status***					
Ever smoked	184	53.2	209	56.3	0.40
Never smoked	162	46.8	162	43.7	
Missing	52	-	1	-	

***Family History***					
Yes^‡^	86	21.6	34	9.2	<10^-5^
No	312	78.4	337	90.8	
Missing	0	-	1	-	

***Menopausal status***					
Pre-menopausal	296	84.0	261	82.1	0.42
Post-menopausal	56	16.0	57	17.9	
Missing^†^	46	-	54	-	

***Age at menarche***					
Age ≤ 12	154	45.2	177	48.1	0.43
Age > 12	187	54.8	191	51.9	
Missing	57	-	4	-	

***Age period stopped***					
Pre-menopausal	296	84.6	261	82.1	0.68
Age ≤ 49	39	11.1	42	13.2	
Age > 49	15	4.3	15	4.7	
Missing	48	-	54	-	

***Parity (# of live births)***					
Nulliparous	68	19.6	63	17.0	0.85
1 child	56	16.1	57	15.4	
2 children	145	41.8	157	42.3	
≥ 3 children	78	22.4	94	25.3	
Missing	51	-	1	-	

***Age at 1^*st *^live birth***					
Nulliparous	68	19.7	63	17.0	0.63
≤ 24 years	126	36.4	136	36.7	
> 24 years	152	43.9	172	46.4	
Missing	52	-	1	-	

*Age of first breast cancer diagnosis for cases and age at interview for controls

^†^Uncertain or missing data

^‡ ^Women with first-degree relatives with breast cancer

^# ^χ^2 ^test for categorical variables or Student's t test (equal variance) for continuous variables

**Table 3:** Analysis of individual SNP effects on breast cancer. Estimated odds-ratios (OR) and 95% confidence intervals (CI) associated with each of the 19 polymorphisms selected under co-dominant age-adjusted main effect models (the significant associations are in bold).

**SNP**	**Genotype**	**Cases n (%)**	**Controls n (%)**	OR*	**95% CI**
**COMT **	AA	94 (23.6)	96 (25.8)	1.0.	-
Met108/158Val	*AG*	202 (50.8)	196 (52.7)	1.3	(0.9–1.9)
	GG	102 (25.6)	80 (21.5)	1.3	(0.9–2.0)

**CCND1 **	*GG*	104 (26.1)	114 (30.6)	1.0.	-
Pro241Pro	*AG*	203 (51.0)	178 (47.8)	1.3	(0.9–1.9)
	AA	91 (22.9)	80 (21.5)	1.3	(0.9–1.8)

**IL13**	*GG*	240 (60.3)	252 (67.8)	1.0.	-
Arg130Gln	*AG*	144 (36.2)	105 (28.2)	0.8	(0.4–1.8)
	*AA*	14 (3.5)	15 (4.0)	1.2	(0.9–1.6)

**IL1A **	GG	204 (51.3)	179 (48.1)	1.0.	-
Ala114Ser	*GT*	155 (38.9)	164 (44.1)	1.0	(0.7–1.3)
	TT	39 (9.8)	29 (7.8)	1.7	(1.0–2.8)

**G-CSF **	*AA*	146 (36.7)	138 (37.1)	1.0.	-
Leu185Leu	*AG*	193 (48.5)	182 (48.9)	1.0	(0.7–1.3)
	*GG*	59 (14.8)	52 (14.0)	1.1	(0.7–1.8)

**ESR1**	*TT*	107 (26.9)	97 (26.1)	1.0.	-
Ser10Ser	*CT*	200 (50.3)	187 (50.3)	1.1	(0.7–1.6)
	*CC*	91 (22.9)	88 (23.7)	1.1	(0.7–1.4)

**TNF-A **	*GG*	274 (68.8)	237 (63.7)	1.0.	-
G(-308)A	*AG*	113 (28.4)	120 (32.2)	0.9	(0.4–2.1)
	*AA*	11 (2.8)	15 (4.0)	0.9	(0.7–1.2)

**CYP17 **	*TT*	170 (42.7)	172 (46.2)	1.0.	-
C(518)T	*CC*	60 (15.1)	49 (13.2)	1.5	(1.0–2.4)
	*CT*	168 (42.2)	151 (40.6)	1.3	(0.9–1.7)

**BARD1 **	*CC*	174 (43.7)	142 (38.2)	1.0.	-
Pro24Ser	*CT*	188 (47.2)	184 (49.5)	0.8	(0.6–1.1)
	*TT*	36 (9.0)	46 (12.4)	0.8	(0.5–1.2)

**XPD **	*AA*	146 (36.7)	165 (44.3)	1.0.	-
Lys751Gln	*AC*	194 (48.7)	167 (44.9)	1.3	(0.9–1.7)
	***CC***	**58 (14.6)**	**40 (10.8)**	**1.6**	**(1.0–2.6)**

**IL10 **	*AA*	90 (22.6)	107 (28.7)	1.0.	-
G(-1082)A	*AG*	205 (51.5)	194 (52.2)	1.1	(0.7–1.5)
	*GG*	103 (25.9)	71 (19.1)	1.1	(0.7–1.6)

**ESR1 **	*CC*	243 (61.1)	213 (57.3)	1.0.	-
Pro325Pro	*CG*	126 (31.7)	138 (37.1)	1.0	(0.7–1.3)
	*GG*	29 (7.3)	21 (5.6)	1.0	(0.5–1.8)

**MTHFR **	*CC*	162 (40.7)	160 (43.0)	1.0.	-
Ala222Val	*CT*	170 (42.7)	161 (43.3)	1.1	(0.8–1.5)
	*TT*	66 (16.6)	51 (13.7)	1.3	(0.8–2.0)

**GSTP1**	*AA*	208 (52.3)	175 (47.0)	1.0.	-
Ile105Val	*AG*	149 (37.4)	161 (43.3)	1.1	(0.8–1.5)
	*GG*	41 (10.3)	36 (9.7)	1.3	(0.8–2.0)

**GADD45 **	*TT*	189 (47.5)	177 (47.6)	1.0.	-
C(IVS3+168)T	*CC*	32 (8.0)	43 (11.6)	0.8	(0.5–1.3)
	*CT*	177 (44.5)	152 (40.9)	0.9	(0.7–1.2)

**P27 **	*TT*	256 (64.3)	235 (63.2)	1.0.	-
Val109Gly	*GG*	17 (4.3)	15 (4.0)	1.0	(0.5–2.0)
	*GT*	125 (31.4)	122 (32.8)	1.0	(0.7–1.3)

**MMP1 **	*Del/del*	110 (27.6)	104 (28.0)	1.0.	-
1G(-1607)2G	*Ins/ins*	92 (23.1)	94 (22.6)	0.8	(0.6–1.3)
	*Ins/del*	196 (49.2)	184 (49.5)	1.0	(0.7–1.4)

**PTEN **	*Del/del*	171 (43.0)	196 (52.7)	1.0.	-
(IVS4+109) ins/delACTAA	*Ins/ins*	47 (11.8)	38 (10.2)	1.3	(0.8–2.1)
	*Ins/del*	180 (45.2)	138 (37.1)	1.2	(0.9–1.7)

**GSTM3 **	*Ins/ins*	271 (68.1)	272 (73.1)	1.0.	-
4595 (3bp ins/del)	*Del/del*	7 (1.8)	9 (2.4)	0.9	(0.4–2.1)
	*Ins/del*	120 (30.1)	91 (24.5)	0.9	(0.7–1.3)

*Age-adjusted odds ratios from unconditional logistic regression analyses.

**Table 4:** Analysis of two-way SNP interaction effects on breast cancer.

Two-way Interactions between polymorphisms	*Crude P-values*^‡^	Bootstrap frequency of stepwise variable selection*	*Bootstrap P-values*^†^	*FDR adjusted P-values*^§^	*FPRP*^¶^
XPD-[Lys751Gln] and IL10-[G(-1082)A]	*0.035*	*68%*	*0.001*	***0.007***	***0.092***
COMT-[Met108/158Val] and CCND1-[Pro241Pro]	*0.010*	*61%*	*0.002*	***0.014***	***0.169***
GSTP1-[Ile105Val] and COMT-[Met108/158Val]	*0.036*	*54%*	*0.001*	***0.007***	***0.093***
CYP17-[C(518)T] and GADD45-[C(IVS3+168)T]	*0.024*	*53%*	*0.018*	*0.062*	*0.999*
BARD1-[Pro24Ser] and ESR1-[Pro325Pro]	*0.039*	*51%*	*ns*^||^	*ns*	-
TNFA-[G(-308)A] and p27-[Val109Gly]	*0.016*	*49%*	*0.025*	***0.079***	*0.996*
BARD1-[Pro24Ser] and p27-[Val109Gly]	*0.021*	*44%*	*ns*	*ns*	-
BARD1-[Pro24Ser] and XPD-[Lys751Gln]	*0.024*	*36%*	*0.002*	***0.014***	*0.671*
ESR1-[Ser10Ser] and ESR1-[Pro325Pro]	*0.028*	*30%*	*0.097*	*ns*	*0.999*

^‡ ^Probability to enter the model in the stepwise multivariable model based on the score statistic in the original data set.

*Proportion of times the interaction is selected by the stepwise multivariable model in the 1,000 bootstrap samples.

^†^*P*-values estimated by bootstrap analysis in the 1,000 random samples.

^§^False Discovery Rate (FDR) adjusted *P*-values.

^||^ns: p > 0.10.

^¶^False Positive Report Probability

**Table 5:** Estimated odds-ratios (ORs) and 95% confidence intervals (CIs) associated with the genotype combinations of the four significant two-way SNP-SNP interactions.

**SNP-SNP interaction**	**SNP Genotypes**		**Cases n**	**Controls n**	**OR**^†^	**95% CI**^†^
	**XPD**	**IL10**				

XPD-[Lys751Gln] and IL10-[G(-1082)A]	*AA*	*AA*	40	57	1.0	-
	*AC*	*AA*	45	39	1.7	[0.9–2.7]
	*CC*	*AA*	21	8	3.7	[1.6–9.7]
	*AA*	*AG*	74	89	1.2	[0.8–1.9]
	*AC*	*AG*	99	71	1.9	[1.3–3.1]
	*CC*	*AG*	22	22	1.4	[0.8–3.1]
	*AA*	*GG*	41	26	2.2	[1.3–3.9]
	*AC*	*GG*	42	50	1.2	[0.7–2.4]
	*CC*	*GG*	14	10	2.2	[0.8–5.5]

	**BARD1**	**XPD**				

BARD1-[Pro24Ser] and XPD-[Lys751Gln]	*CC*	*AA*	66	60	1.0	-
	*CT*	*AA*	68	90	0.7	[0.4–1.1]
	*TT*	*AA*	21	22	0.9	[0.4–1.8]
	*CC*	*AC*	82	67	1.1	[0.7–1.8]
	*CT*	*AC*	89	69	1.2	[0.7–1.9]
	*TT*	*AC*	15	24	0.6	[0.3–1.2]
	*CC*	*CC*	23	13	1.6	[0.7–3.4]
	*CT*	*CC*	27	26	1.0	[0.5–1.9]
	*TT*	*CC*	7	1	7.4	[1.3–12.4]

	**COMT**	**CCND1**				

COMT-[Met108/158Val] and CCND1-[Pro241Pro]	*AA*	*GG*	21	40	1.0	-
	*AG*	*GG*	57	53	1.9	[1.3–4.6]
	*GG*	*GG*	25	21	2.4	[1.0–4.9]
	*AA*	*AG*	48	36	2.6	[1.3–4.5]
	*AG*	*AG*	110	92	2.3	[1.3–4.5]
	*GG*	*AG*	38	45	1.6	[1.0–3.2]
	*AA*	*AA*	22	29	1.4	[0.7–3.3]
	*AG*	*AA*	43	37	2.2	[1.1–4.3]
	*GG*	*AA*	34	19	3.3	[1.9–7.2]

	**GSTP1**	**COMT**				

GSTP1-[Ile105Val] and COMT-[Met108/158Val]	*AA*	*AA*	30	49	1.0	-
	*AG*	*AA*	46	48	1.5	[0.9–2.8]
	*GG*	*AA*	15	8	3.4	[1.1–11.4]
	*AA*	*AG*	95	92	1.7	[1.0–2.6]
	*AG*	*AG*	91	69	2.2	[1.4–3.6]
	*GG*	*AG*	24	21	1.8	[0.9–4.8]
	*AA*	*GG*	54	36	2.6	[1.4–4.2]
	*AG*	*GG*	36	41	1.5	[0.9–2.7]
	*GG*	*GG*	7	8	1.6	[0.5–4.2]

^†^OR and CIs estimated by bootstrap analysis in the 1,000 random samples.
